# Outcome, quality of life and direct costs after out-of-hospital cardiac arrest in an urban region of Switzerland

**DOI:** 10.1186/s13049-019-0682-7

**Published:** 2019-11-27

**Authors:** Raphael Ruch, Laura Stoessel, Philipp Stein, Michael Thomas Ganter, Daniel Anthony Button

**Affiliations:** Institute of Anesthesiology – Emergency Medical Service, Perioperative Medicine, Pain Therapy, Kantonsspital Winterthur, Brauerstrasse 15, CH-8401 Winterthur, Switzerland

**Keywords:** Out-of-hospital cardiac arrest, Resuscitation, Quality of life, Hospital cost

## Abstract

**Background:**

Considering the significant morbidity and mortality of out-of-hospital cardiac arrest, only little data on survival or quality of life after successful resuscitation is available in Europe. Additionally, economic aspects of such events are poorly studied. The purpose of this study is to provide data for survival, quality of life and costs directly related to the cardiac arrest for a region of Switzerland served by one emergency medical service (EMS).

**Methods:**

Eighty eight patients older than 18 years of age that were resuscitated by the EMS Winterthur in the year 2013 were included and retrospective analysis of EMS-protocols was performed. For patients alive at follow-up, 2 years after the event, a structured interview with quality of life questionnaires was conducted. This study was accepted by the local Ethics Committee.

**Results:**

Thirty five percent (*n* = 31) of resuscitated patients were admitted alive to the hospital following out-of-hospital cardiac arrest. This incidence was as high as 60%, if the patients had a shockable rhythm as first rhythm. Survival to follow-up was 16% (*n* = 14). These patients had an excellent quality of life overall, with little to no limitations in daily life. There was no significant difference in survival for patients in outlying regions with comparatively longer timespans until arrival of EMS. Median EMS-costs for deceased patients were CHF 1731 (inter-quartile range 346), for survivors CHF 2′169 (inter-quartile range CHF 444) and median hospital-costs were CHF 27′707 (inter-quartile range CHF 62′783).

**Conclusion:**

Quality of care for patients with out-of-hospital cardiac arrest in the region of Winterthur is high, including patients in outlying regions. The associated costs are similar to other European countries.

**Trial registration:**

This trial was registered with www.clinicaltrials.gov under NCT02625883.

## Background

Even though unexpected out-of-hospital cardiac arrest (OHCA) is a disastrous event with significant morbidity and mortality, data on survival after OHCA in Switzerland and even Europe is sparse [1, 2]. In terms of creating evidence by performing prospective randomized studies, only one such trial was published per year in German-speaking Europe between 1990 and 2012 on average [3]. In Switzerland, some data on outcome after OHCA have been published, however, routine reporting is not available although this could serve as an important quality marker for prehospital clinical care [4–6]. Studies on quality of life (QoL) are seldom and usually focus on neurological deficit instead of structured and validated QoL-scores [7]. In Switzerland, no analysis of QoL after OHCA exists so far. Additionally, little data is available concerning costs associated with treatment of patients with OHCA. Since resources are limited in our modern health care systems, economic aspects including costs gather more and more attention [8].

The *first aim* of the present study was to retrospectively analyze the survival after OHCA in the emergency medical service (EMS) Winterthur (“Rettungsdienst Winterthur”) in 2013. The *second aim* was to assess QoL 2 years after OHCA. Finally, the *third aim* was to calculate health care costs from OHCA until hospital discharge of this patient population.

## Methods

This study was approved by the local Ethics Committee of Zurich, Switzerland (KEK ZH 2015–0396) and registered at clinicaltrials.gov (NCT02625883). The need for informed consent of family members of deceased patients was waived, informed consent of surviving patients was obtained.

Our study was a combination of a retrospective analysis, based on EMS Winterthur data from the year 2013, and a structured follow up interview 2 years later with all surviving patients from that data set. All adult patients suffering from OHCA requiring resuscitation by the EMS Winterthur were included. Exclusion criteria were age < 18 years old and cardiac arrest other than cardiac origin (e.g. trauma, submersion, drug overdose, asphyxia, exsanguination). In addition, patients who denied the use of their data for research purposes and patients with incomplete datasets were not included in data analysis.

Data for OHCA was collected according to Utstein criteria [9, 10]. Location and address of the cardiac arrest was documented. Initial cardiac rhythm was recorded and classified as shockable and non-shockable. Per Utstein criteria, return of spontaneous circulation (ROSC) and/or termination of prehospital cardiopulmonary resuscitation (CPR) were encoded to the database. ROSC was defined as at least a brief (> 30 s) restoration of a spontaneous perfusing rhythm, that provides evidence of more than an occasional gasp, fleeting palpated pulse or arterial waveform according to the Utstein criteria [9, 10]. Only patients with sustained ROSC were transported to the hospital, otherwise CPR was terminated in the field.

Our primary endpoint was the incidence of sustained ROSC and 2-year survival after OHCA. Second endpoint was QoL at least 2 years after the event. Third endpoint was costs generated by the health care system until first discharge in the care for patients with ROSC after resuscitation for OHCA.

All EMS in the canton Zurich are dispatched by a central emergency call center located at the Zurich airport. In cases of reported cardiac arrest, the dispatcher instructs callers to perform CPR. Although this so-called telephone-CPR has been institutionalized for many years, structured documentation of telephone-CPR has just started recently and was not established at the time of the study. The EMS Winterthur serves the northern part of the canton Zurich (around 250′000 inhabitants) including the city of Winterthur. It consists of 55 full-time employees, 7 ambulance vehicles and 2 emergency physician transport vehicles and is dispatched to about 25 emergency calls daily, around 9′100 per year. The resuscitation is performed according to current advanced life support (ALS) guidelines by the European Resuscitation Council. An emergency physician can be mobilized by paramedics already on site or may accompany the ambulance crew from the beginning, depending on the severity of the reported emergency. Additionally, there exists a countrywide physician-staffed, helicopter-based EMS for calls to more remote or mountainous regions. None of our analysed cases was handled by these crews, however.

### Survival and quality of life

Data sources included Utstein and operational protocols including timestamps from EMS Winterthur and – for surviving patients – medical records from hospitals and rehabilitation clinics.

Data on QoL was collected using the subjective classification with the Cerebral Performance Category (CPC) [11], the EQ-5D-5 L-questionnaire (EuroQol, The Netherlands) [12] and a 36-item health survey (RAND Health Care, RAND Corporation, Santa Monica, CA).

Although not validated, the easy to use CPC-scale is widely being used to assess neurological impairment and provide reliable information on functional outcome categories [13]. CPC-scores range from 1 to 5, scores 1 to 3 indicating no, mild or severe cerebral damage, 4 representing coma or a vegetative state and 5 brain death.

The EQ-5D-5 L-questionnaire is a validated QoL-score analyzing 5 dimensions of health, mobility, self-care, usual activities, pain/discomfort, anxiety/depression in 5 levels from 1 to 5, representing no problems, slight, moderate, severe problems or inability to perform, respectively. Additionally, it includes a visual analog scale for general health ranging from 0 to 100. It is available in a German translation, so it can be safely used in our German speaking population. Although standard values for Switzerland do not exist, the score has been used to determine standard values for many European countries, most of them with a health care standard comparable to Switzerland [14, 15]. These population norms were collected for the 3-level version of the questionnaire, they can however be used for the 5-level-version as well [16].

The RAND 36-item health survey was added as an additional, more extensive, tool to assess QoL. Its 36 questions are merged into a percentile rank in eight different health-related dimensions, physical functioning, role limitations due to physical health, role limitations due to emotional problems, energy/fatigue, emotional well-being, social functioning, pain and general health.

### Financial aspects

For patients with ROSC, we considered the invoice-totals (direct costs) from EMS and hospitals until first discharged.

### Statistical analysis

Statistical analysis was performed with R version 3.5.0, published under the GNU general public license (The R foundation for statistical computing, www.r-project.org, Vienna, Austria, 2018). Methods used were linear regression with least squares for continuous data, Student’s t-test for group-comparison and Fisher’s exact test for nominal data. Data is shown as median and interquartile range (IQR) and mean ± standard deviation (SD) if not displayed differently. The significance threshold was set at .05.

## Results

**Fig. 1:**
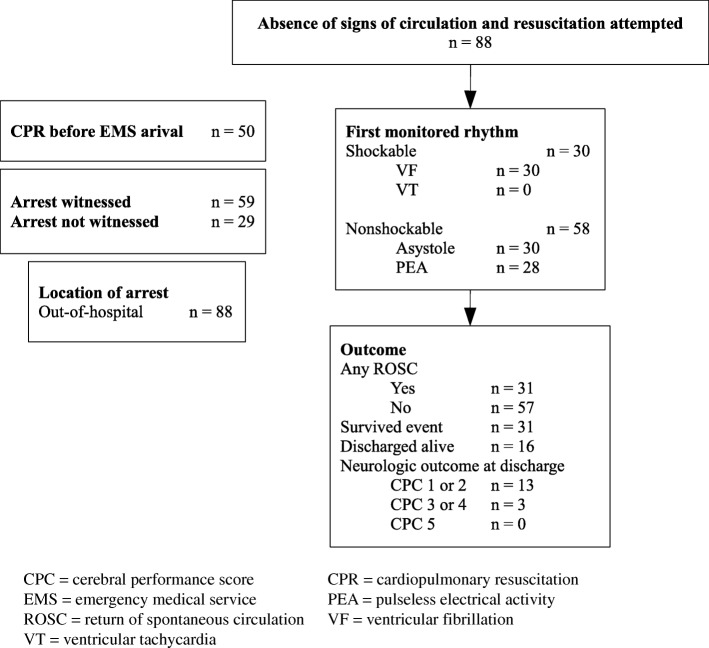
Utstein style flowchart of our patient collective

A total of 91 patients were resuscitated by the EMS Winterthur due to cardiac arrest in 2013. Of them, three individuals were excluded due to missing initial cardiac rhythm. Demographic data and baseline characteristics of the 88 patients studied is shown in Table 1 and as an Utstein style flowchart in Fig. 1.

**Table 1:** Descriptive statistics of our collective

Total number of patients, n	88
Female, n (%)	27 (31)
Mean age, years	68 ± 17
*Initial rhythm*	
Ventricular fibrillation, n (%)	30 (34)
Ventricular tachycardia, n (%)	0 (0)
Pulseless electrical activity, n (%)	28 (32)
Asystole, n (%)	30 (34)
ROSC, n (%)	31 (35)
Collapse witnessed, n (%)	59 (67)
Bystander resuscitation, n (%)	50 (57)
Distance to hospital, km	7 ± 6
*Time parameters*	
Time from EMS activation to arrival on scene, min	9 ± 4
Time from arrival to defibrillation, min	7 ± 6
Time from arrival to ROSC, min	19 ± 11
Time from arrival to discontinuation of resuscitation efforts, min	20 ± 14
*Therapy*	
Defibrillation, n (%)	35 (40)
Epinephrine administered, % n (%)	55 (62)
Amiodarone administered, n (%)	16 (18)

Results are presented as n (percentage of population) or mean ± standard deviation respectively; *EMS* emergency medical service, *ROSC* return of spontaneous circulation

### Survival

Follow-up examination was done at the end of the year 2015, in median 2 years and 7.5 months after the OHCA event (IQR 6.5 months).

Of the overall 88 patients included in data analysis, 35% (*n* = 31) had sustained ROSC and survived to hospital admission, 18% (*n* = 16) were alive at hospital discharge and 16% (*n* = 14) were still alive at follow-up. Thirty of those 88 patients had a shockable initial rhythm (ventricular fibrillation, VF). The outcomes of this subgroup were as follows: 60% (*n* = 18) had sustained ROSC and survived to hospital admission, 47% (n = 14) were alive at hospital discharge and 43% (*n* = 13) were still alive at follow-up.

Patients with ROSC mostly had VF and pulseless electrical activity (PEA) as initial rhythm (VF: 58%, PEA 39%, asystole 3%), while asystole was predominant in patients without ROSC (VF: 21%, PEA 28%, asystole: 51%). The higher rate of ROSC was significant for VF and PEA compared to asystole (*p* < .005 for both), however there was no significant difference between VF and PEA (*p* = .29). Additionally, ROSC could be established more often, if the collapse was witnessed (90% vs 54%, p < .005). The percentage of bystander resuscitation was similar in both groups with 52% in the ROSC-group and 60% in the non-ROSC-group (*p* = .5).

**Fig. 2:**
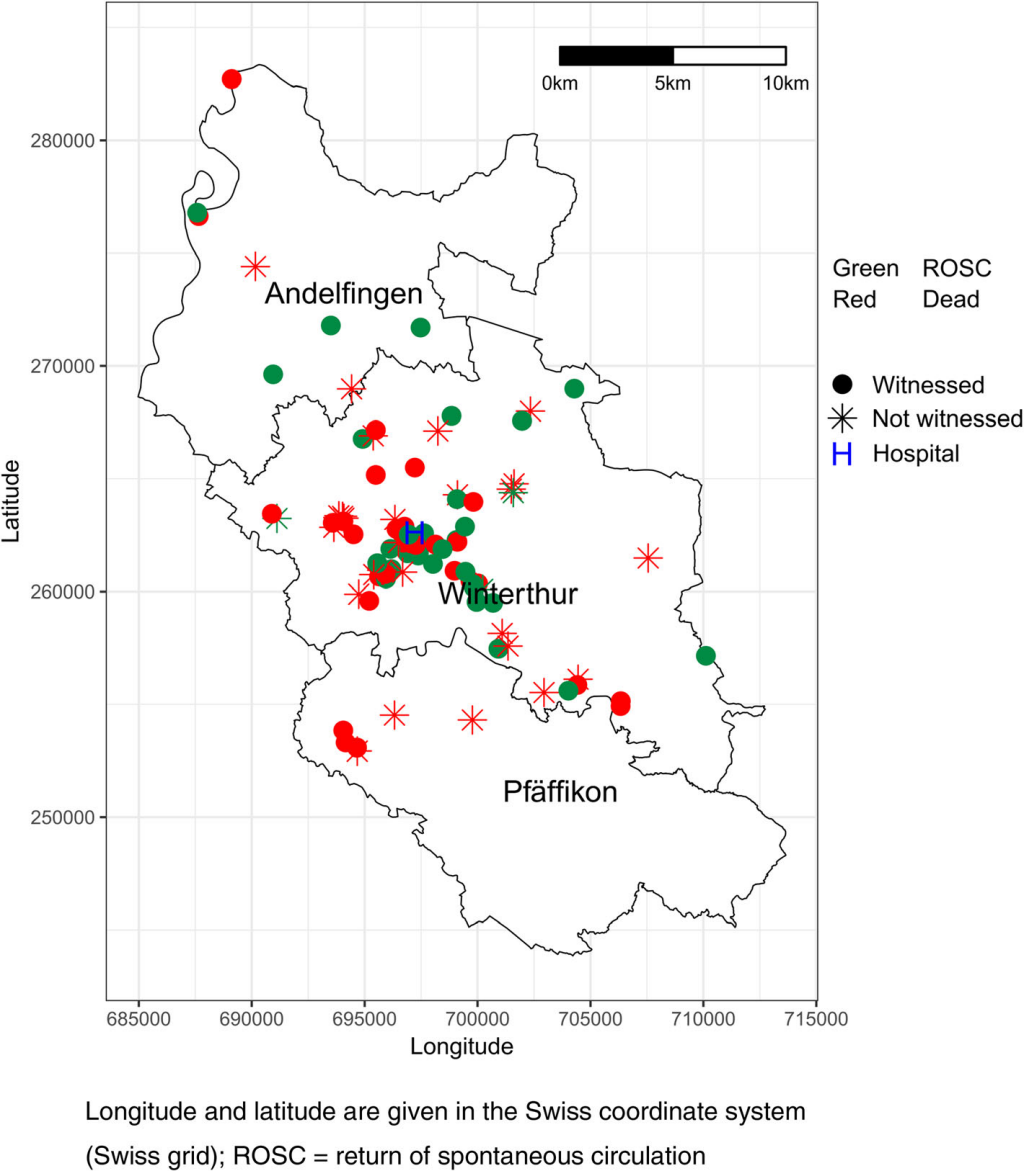
Distribution of out-of-hospital resuscitation by the emergency medical service Winterthur in 2013

Between the ROSC and non-ROSC-group there was no significant difference in the mean distance to the hospital (6 ± 4 km vs 8 ± 4 km, *p* = .2) and mean time from alarm to arrival of EMS on scene (8 ± 6 min vs 9 ± 6 min, *p* = .1). Figure 2 shows the distribution of cases over the area of operations of EMS Winterthur.

The ROSC-group (compared to non-ROSC-group) had a higher incidence of a shockable rhythm (61% vs 28%, *p* < .005). There was no significant difference in the mean dose of epinephrine administered between patients with ROSC or no ROSC (1.9 mg vs 1.8 mg, *p* = .9), nor depending on initial rhythm (VF: 2.2 mg, PEA: 1.7 mg, asystole: 1.5 mg; VF vs PEA: *p* = .4; VF vs asystole: *p* = .3; PEA vs asystole: *p* = .7).

### Quality of life

Of the 14 patients alive at follow-up, one was in critical condition and died before filling in the questionnaire and another did not consent. Thus, we conducted interviews with 12 patients.

Neurological outcome in all surviving patients was good, 11 patients were assigned a CPC-score of 1 (no cerebral damage). Only one patient had a CPC-score of 2 (mild cerebral damage). The results of the EQ-5D-5 L questionnaire and the RAND 36-item health survey are given in Table 2.

**Table 2:** Results of the EQ-5D-5 L questionnaire and RAND 36-item health survey

Category	Score
*EQ-5D-5 L*	
Mobility	1.5 (1 / 3)
Self-Care	1 (1 / 1)
Usual activities	1 (1 / 2)
Pain/discomfort	1 (1 / 3)
Anxiety/depression	1 (1 / 3)
VAS	87.5 (50 / 100)
*RAND 36*	
Physical functioning	95 (55 / 100)
Role limitations (due to physical health)	100 (0 / 100)
Role limitations (due to emotional problems)	100 (0 / 100)
Energy/fatigue	70 (25 / 85)
Emotional well-being	80 (32 / 96)
Social functioning	100 (62.5 / 100)
Pain	100 (32.5 / 100)
General Health	77.5 (40 / 90)

Results are shown as median (min/max). EQ-5D-5 L (except for VAS): score-range 1–5, 1 = no problems, 5 = extreme problems, VAS = visual analog scale for general health, 0–100. RAND 36: percentile score, a lower score means more limitations/problems

Of the 12 patients that could be interviewed at follow-up, only 1 had a PEA as initial rhythm. The initial rhythm of all other patients who were still alive and able to conduct the interview was VF. The only patient with a CPC-score of 2 was in the group of VF.

Table 3 presents the data for QoL in a different manner to enable comparison of our results, using population norms for EQ-5D, which are available for the 3-level version of the questionnaire [14, 16]. Since whole-population responses usually include very few people reporting any problems at all, the results are essentially reported in percentage of people with a score higher than one, thus converting the 3- or 5-level dimensions into 2-level dimensions (problems vs no problems). Of the available population norms, Netherlands’ and Belgium’s health system seem most comparable to Switzerland [15] and are thus added in Table 3.

**Table 3:** Population standard values for the EQ-5D-5 L questionnaire for Belgium and the Netherlands [14] compared to our results from the emergency medical service (EMS) Winterthur

Category	EMS Winterthur	Belgium	Netherlands
Mobility, %	50	12.6	11.5
Self-Care, %	0	4.0	3.4
Usual activities, %	17	12.4	13.5
Pain/discomfort, %	17	28.5	34.2
Anxiety/depression, %	33	6.6	3.5
VAS	87.5	71.3	78.0

Other than VAS, numbers show percentage of people reporting problems (by marking a score higher than 1); VAS norms are given for the age group of 65 – 74y, VAS = visual analog scale for general health, 0–100

### Financial aspects

Length of stay in hospital, EMS costs and hospital costs are given in Table 4. If patients had ROSC, EMS costs were significantly higher than for patients without ROSC (*p* < 0.005). Hospital costs increased significantly with length of stay in the hospital (*p* < .005). There was however no significant difference with regard to survival to discharge from hospital for either (*p* = .14). Also, there was no significant difference in costs depending on whether or not the initial rhythm was shockable (*p* = .46).

**Table 4:** Costs for emergency medical service and hospital and length of stay in hospital (LOS)

*All patients without ROSC*	
EMS costs, CHF	1731 (795/2458)
*All patients with ROSC*	
LOS, days	10 (1/58)
EMS costs, CHF	2′168 (1′262/2′704)
Hospital costs, CHF	28′079 (2′296/330′337)
*Patients with ROSC surviving to discharge*	
LOS, days	19 (5/58)
EMS costs, CHF	2′101 (1′700/2′704)
Hospital costs, CHF	32′909 (3′802/330′337)
*Patients with ROSC not surviving to discharge*	
LOS, days	5 (1/47)
EMS costs, CHF	2′212 (1′262/2′636)
Hospital costs, CHF	27′335 (2′296/104′435)

Results are shown as median (min/max)

## Discussion

Our ROSC-rate of 35% is higher than the 25% the EuReCa ONE-study found for an European average [2]. However, ROSC-rate was equal to the 32% found in a recent study in our neighboring city of Zurich, Switzerland after widely training all Zurich police forces to provide basic life support and equipping all police cars with automated external defibrillators [4]. Additionally, all surviving patients reported good neurological outcomes as well as overall good QoL compared with population norms, as found in multiple other QoL-studies [7, 17, 18].

### Survival

Looking at the rate of survival to discharge, our 18% compare to the 13.2% found in an Australian study [18]. A study concerning the Swiss canton of Ticino found survival rates of 56% to hospital and 24% at 1 year, they however only considered patients presenting with shockable initial rhythms [5], for this subgroup, our percentage rises to 60% survival to hospital and 43% alive at follow-up. A higher survival rate for patients with VF was also found in the EuReCa ONE-study [2].

These results seem to indicate a high quality of care. A contributing factor for a higher percentage of survival might be the predominantly urban area of operations of EMS Winterthur effecting relatively short timespans from alarm to EMS-arrival on scene.

Bystander resuscitation was 52%, slightly lower than the 57% found by Mauri et al. [5] but higher than the 42% found by Sauter et al. [6] and the 47% average in Europe [2].

Distance to hospital not being a factor in survival or ROSC is somewhat surprising, however the similarity in time from alarm to arrival of EMS on scene for ROSC- vs. non-ROSC-group suggests that there existed no significant time difference either to resuscitation or – if bystander resuscitation was performed – to transfer of resuscitation-efforts to EMS.

In our study, although the study sample was small, the administration of epinephrine neither had a positive effect on the survival-rate, nor did it impair neurological outcomes contrary to a recent randomized trial [19].

### Quality of life

It has been claimed that full neurological recovery in patients with out-of-hospital cardiac arrest and shockable initial cardiac rhythm should at least reach 30% [20]. This number can serve as quality indicator for EMS, which has been well achieved by the EMS Winterthur.

Our good neurological outcome – all patients reporting CPC-scores of 1 or 2 – is also reflected in the EQ-5D-5 L scores, where no patients reported any problems with self-care. Table 3 shows a higher incidence of problems in the mobility- and anxiety/depression-dimensions for our collective compared with population standard values from Belgium and the Netherlands. This is probably attributable to the higher age of our patient collective. The other values are well within expected population standard values [14].

The RAND 36-item health survey showed pronounced role limitations due to physical health for one patient and due to emotional problems for 2 patients. This is not reflected in CPC or EQ-5D-5 L and might be an overestimation due to the limited differentiation in the RAND-questionnaire, offering only 3 levels of discrimination for the respective questions.

### Financial aspects

Analysis of costs with regards to neurological outcome was not possible, with only one patient having a differing CPC-score.

EMS-costs for resuscitated patients was negligible compared to hospital-costs. As most post-resuscitation-patients spend at least some time in an intensive care unit with costly infrastructure and expensive personnel, this comes as no surprise. These investments also explain why length of stay in hospital is the main-factor determining hospital-costs.

As financial data for Switzerland is missing, we need to compare our median costs of CHF 28′079 with other countries. Studies in Japan and the Netherlands found similar values [21, 22], while Finland, the UK and Germany reported higher numbers [17, 23, 24].

According to a study comparing hospital-costs for treating ST-elevation myocardial infarction with care after OHCA, the financial parameters for these two entities were similar [25].

### Limitations

This study has several limitations. First, the number of patients is relatively small. However, this data shows that our prehospital care system including first responders has a high standard of care and is at least comparable to other published European centers. Second, our results describe the survival rate and QoL of a specific population served by one EMS only and therefore may not automatically be applicable to other EMS systems.

In 2 instances the ambulance dispatched to the OHCA-case was not departing from the EMS-headquarters but rerouted on return from another assignment. This may influence the registered distance from the hospital to the site of the OHCA-event.

## Conclusion

This is one of the first Swiss studies to collectively investigate outcome, quality of life and financial aspects in out-of-hospital resuscitation. Quality of care for patients with out-of-hospital cardiac arrest in Winterthur seems to conform to European standards. Survival being independent of distance to hospital suggests a high standard of care by first responders covering outlying regions. While QoL for patients with OHCA seems to be very good, further research, especially using larger case numbers, could focus on QoL after OHCA, identifying specific problem areas in QoL. Potential therapeutic measures could then be evaluated to improve on these main causes of lost QoL. In addition, more research into financial aspects of OHCA is needed, focusing on direct costs (hospital, EMS and rehabilitation) as well as indirect costs (absence at work due to illness, loss of productive life-years).

## Data Availability

The dataset analysed during the current study is available from the corresponding author on reasonable request.

## Abbreviations

ALS: advanced life support
CPC: cerebral performance score
CPR: cardiopulmonary resuscitation
EMS: emergency medical service
IQR: inter-quartile range
LOS: length of stay
OHCA: out-of-hospital cardiac arrest
PEA: pulseless electrical activity
QoL: quality of life
ROSC: return of spontaneous circulation
SD: standard deviation
VAS: visual analog scale
VF: ventricular fibrillation
